# Functional characterization of a common XIa inhibitory Kunitz-domain Shp4 from nine schistosoma secreted proteins with diverse C-terminal fragments

**DOI:** 10.1371/journal.pntd.0013282

**Published:** 2025-07-08

**Authors:** Xiangdong Ye, Xiaolin Deng, Huanhuan Gao, Shuwen Chen, Li Ding, Fang Sun, Chenhu Qin, Hongyi Luo, Wen Zhu, Xudong Luo, Zongyun Chen

**Affiliations:** 1 Institute of Biomedicine, Hubei Key Laboratory of Embryonic Stem Cell Research and Hubei Key Laboratory of Wudang Local Chinese Medicine Research, College of Basic Medicine, Hubei University of Medicine, Shiyan, China; 2 Hubei Key Laboratory of Wudang Local Chinese Medicine Research, College of Basic Medicine, Hubei University of Medicine, Shiyan, China; 3 Department of Clinical Laboratory, Dongfeng Hospital, Hubei University of Medicine, Shiyan, China; IRNASA, CSIC, SPAIN

## Abstract

Coagulation factor XIa is a drug target for new anticoagulants, but no XIa inhibitors have been found from schistosoma, a worm living in the bloodstream. In this study, by sequence blasting and structural fold searching of Kunitz-domain containing proteins from schistosoma, a specific Kunitz-domain Shp4 that appears in nine secreted proteins from four schistosoma species was identified successfully. Functional studies showed that Shp4 is a novel XIa inhibitor with a Ki value of 3.35 nM, which inhibited coagulation factor XIa activity in a non-heparin-dependent manner. His-pull-down assay further indicated the direct interaction between Shp4 and XIa. Animal experiments showed that Shp4 is a useful peptide lead drug with well antithrombotic effects *in vivo*. Alanine-scanning experiments showed that R18 is the key residue for XIa inhibitory and anticoagulation activities. Structural modelling of nine schistosoma-derived full-length secreted proteins suggested that the C-terminal non-Kunitz-domain fragments might play important roles in modulating the Kunitz-domain functions by steric hindrance effect and transmembrane helix structure. In conclusion, our work characterized the first XIa inhibitor from schistosoma with high anticoagulation activity and well antithrombotic effects, and highlights a potential strategy to modulate the Kunitz-domain, not only by the functional loop, but also by diverse C-terminal fragments.

## Author summary

Schistosoma has a unique parasite life cycle that lives in the veins of the mammalian host and feeds on its blood, which suggests that schistosoma might be a natural source of novel anticoagulants that can balance the coagulation and anticoagulation systems. Here, by sequence blasting and structural fold searching of Kunitz-domain containing proteins from schistosoma, a specific Kunitz-domain Shp4 that appears in nine secreted proteins from four schistosoma species was identified successfully. We found that Shp4 had potent XIa inhibitory activity and well antithrombotic effects. Besides this, by structural modelling of nine schistosoma-derived secreted proteins, we firstly found that the C-terminal non-Kunitz-domain fragments might play important roles in modulating the Kunitz-domain protease-inhibitory activities by steric hindrance effect and transmembrane helix structure. In conclusion, our work not only characterized the first XIa inhibitor from schistosoma with high anticoagulation activity and well antithrombotic effects, and but also highlights a potential strategy to discovery new functional Kunitz-domain peptides from medical related species with blooding-sucking life habits.

## 1. Introduction

Since the discovery of heparin and warfarin, research on anticoagulants has continued for nearly 100 years [1–3]. Target-specific anticoagulants used to be a good choice to discover novel anticoagulants and drugs, such as dabigatran targeting coagulation factor thrombin and rivaroxaban, apixaban and edoxaban targeting coagulation factor Xa [4–6]. As we know, coagulation factor XIa is also an important serine protease in the coagulation pathway [7]. In contrast to two classical anticoagulant targets Xa and thrombin, patients with deficiency in plasma Coagulation factor XIa experienced a lower probability of thrombosis associated diseases, such as deep vein thrombosis and ischemic stroke [8,9]. Thus, Coagulation factor XIa has been recognized as a new drug target for antithrombotic drug design in recent years [10–12].

Kunitz-domain peptides are an important natural resource and widely distributed among various species such as animals, plants, microorganisms, etc [13–17], which is an important molecular scaffold for lead drug discovery [18–20]. Till now, two peptides with Kunitz-type structural fold aprotinin and Ecallantide have been approved for clinical use [21,22]. Aprotinin has a wide inhibitory action with particular activity against trypsin, chymotrypsin and kallikrein, making it attractive in ameliorating the effects of acute pancreatitis [23,24]. Ecallantide is a highly active plasma kallikrein inhibitors that block the binding site of kallikrein to prevent cleavage of high molecular weight kininogen and subsequent bradykinin generation, and was shown to be efficacious for hereditary angioedema (HAE) attacks [25]. XIa-inhibitory Kunitz-type peptides have also been found from some blood-sucking and venomous animals, such as Ir-CPI from tick [16,26], and Desmolaris from bat [27], Fasxiator, DAKS1, and BF9 from snake [28–30]. So, it is effective strategy to discovery functional Kunitz-type peptides from diverse biological resources.

Schistosoma is a surprising blood-sucking animal that has a unique parasite life cycle that lives in the veins of the mammalian host and feeds on its blood [31]. This curious phenomenon suggests that schistosoma might be a natural source of novel anticoagulants that can balance the coagulation and anticoagulation systems [31–33]. Three Kunitz-domain containing anticoagulants have been characterized from schistosoma, such as two secreted peptides SjKI-1 and Schixator from *Schistosoma japonicum* and one secreted protein SmKI-1 from *Schistosoma mansoni* [34–36]. However, no XIa inhibitors have been found from schistosoma. Here, by combination with bioinformatics analyses and functional characterization, a common XIa inhibitory Kunitz-domain Shp4 from nine secreted proteins with diverse C-terminal fragments was discovered and characterized [16].

## 2. Materials and methods

### 2.1. Ethics statement

All animal experiments were approved by the Animal Protection and Utilization Committee of the Animal Research Institute of Hubei University of Medicine (Accreditation Code: 2023–060). Animal experiments were performed in accordance with the NIH Guide for the Care and Use of Laboratory Animals.

### 2.2. Bioinformatic analysis

Sequences were identified for open reading frames using ORFfinder (http://www.ncbi.nlm.nih.gov/projects/gorf/). The intron was predicted by the GT-AG splice site rule. The signal peptide was predicted by the SignaIP-5.0 server (http://www.cbs.dtu.dk/services/SignalP/index.php). The transcriptomic profile and genome data of Schistosoma are available at WormBase ParaSites (https://parasite.wormbase.org/index.html/). After excluding signal peptides, the similarity was analyzed by searching against the GenBank NCBI database (http://www.ncbi.nlm.nih.gov/blast) using BLAST algorithms. Molecular weight and isoelectric point calculations were performed using the ExPASy-Compute pI/Mw tool (http://web.expasy.org/compute_pi/). The characteristic Kunitz protein domain was identified by searching the PROSITE database (http://prosite.expasy.org/), and a multiple sequence alignment of the Kunitz-domain peptide from Schistosoma was generated with the Clustal Omega program (http://www.ebi.ac.uk/Tools/msa/clustalo/). The atomic structure of the Kunitz-type peptide was modeled by using boophilin (PDB Code: 2ODY) as a template in the SWISS-MODEL server (https://www.swissmodel.expasy.org/) [37,38].

### 2.3. Recombinant expression and purification of Kunitz-domain peptide

The recombinant plasmid pET-28a-Schistosoma Kunitz peptide Shp4 was constructed by overlapping PCR with optimal codons according to our previous work [39,40]. The recombinant plasmid was verified by DNA sequencing before expression. Schistosoma Kunitz peptide Shp4 was expressed and purified based on our previous work [41–44]. The plasmid pET-28a-Shp4 was transformed into competent *Escherichia coli* BL21 (DE3) cells (Beijing Quanshi Jin Biotech Co., Ltd), and the transformed bacteria were cultured in 1–2 liters of medium. The Schistosoma Kunitz peptide Shp4 with enriched disulfide bridges was found to exclusively accumulate in inclusion bodies. It was refolded in vitro as we have described before [42,45]. The insoluble inclusion bodies were washed twice with washing buffer (1%–2% Triton X-100 in phosphate buffered saline) and denatured in 5 ml denaturation solution (6 M guanidinium-HCl, 0.1 M Tris-HCl pH 8.0, 1 mM EDTA, and 30 mM reduced glutathione). Then, recombinant peptide Shp4 was reactivated by 100-fold dilution in renaturation solutions at approximately pH 8.0 (0.2 M ammonium acetate at pH 8.0, containing 0.2 mM oxidized glutathione and 0.5 M arginine) at 16 °C for 24 h. Renatured peptide Shp4 was finally purified by high-performance liquid chromatography (HPLC) on a C18 column (10 mm × 250 mm, 5 μm, Dalian Elite, China). Peaks were detected at 230 nm. The fraction containing Schistosoma Kunitz peptide Shp4 was eluted at approximately 21 min and collected manually. The Shp4 fraction was immediately lyophilized, and matrix-assisted laser desorption ionization time-of-flight mass spectrometry (MALDI-TOF-MS) was applied to identify the molecular weight of the purified Shp4 peptide. Given that Shp4 is a Kunitz-domain peptide with six conserved cysteines that can form three disulfide bridges, a mass loss of 6 Da from the Cys thiol groups engaged in the disulfide bridges is expected for the correctly folded, oxidized Shp4.

### 2.4. Activated partial thromboplastin time (APTT), prothrombin time (PT) and thrombin time (TT)

We used APTT, PT, and TT kits (Meide Pacific Biotechnology Co., Ltd, Tianjin, China) to detect the anticoagulant function of peptides. The activated partial thromboplastin time (APTT), prothrombin time (PT) and thrombin time (TT) experiments were performed as described before [34]. Clotting was initiated by the addition of 25 mM CaCl_2_ to the mixture, and clot formation was measured using an Infinite M200 microplate reader at 650 nm for fibrin polymer formation.

### 2.5. Serne protease inhibitory activity assay

The inhibitory activity of peptides was tested in the presence of serine proteases and their corresponding fluorogenic substrates. Respective serine protease (25 μL) diluted with buffer (50 mM Tris, pH 7.4, 140 mM NaCl, 5 mM CaCl_2_, 0.1% BSA) was preincubated with 25 μL of peptide for 30 min at 37 °C, followed by the addition of 50 μL of the appropriate chromogenic substrate. In a total volume of 100 μL, the final serine protease/substrate concentrations were as follows: Kallikrein (9.0 nM) from Enzyme Research Laboratories, S2302 (1.6 mM) from Chromogenix, FXIIa (6.7 nM) from Molecular Innovations, S2302 (0.8 mM) from Chromogenix, XIa (0.5 nM) from Hematologic Technologies Inc., S2366 (1.2 mM) from Chromogenix, and thrombin (2 nM) from Hematologic Technologies Inc., S2238 (1.6 mM) from Chromogenix. The other serine proteases and fluorogenic substrates were prepared as we have described before [28,46,47], and all the substrate cleavages were measured at 405 nm using a microplate reader.

### 2.6. His-pull down

His-pull down was used to confirm the binding effect between peptide and serine proteases. The solution containing nickel beads was centrifuged at 4 °C for 5 minutes at 4000 rpm and washed three times with ice-cold TBS. Nickel beads (Changzhou Tiandi Renhe Biotechnology Co., Ltd.) were prepared into a 50% solution by the TBS method, and each tube was 40 μL and named A, B, or C. Then, 30 μg of peptide was added to tubes A and B, and tube C was used as a negative control with no peptide. Then, 500 μL TBS was added to each tube and incubated at 4 °C for 12 h. After centrifugation, the supernatant was discarded, and the precipitate was washed twice with TBST and once with TBS. Then, 10 μg XIa was added to tubes B and C but not to tube A. Then, 500 μL TBS was added to each tube and incubated at 4 °C for 2 h. After washing again, 20 μL 2 × loading was added to the precipitate at 100 °C for 10 minutes. Each supernatant was collected and separated by 15% SDS–PAGE and stained with Coomassie Blue. The same method was used to study the interaction between the peptide and XIa.

### 2.7. Thromboelastography experiment

The thromboelastograph experiment was carried out to test the anticoagulation activity of the peptide in whole blood. First, a normal test cup was loaded in the test channel, and 1 ml of mixed sodium citrate anticoagulant whole blood and 50 µL of diluted peptide with normal saline were added to a kaolin tube. Two different concentrations, 5 μg/mL and 10 μg/mL, of the peptide were chosen and evaluated. The tube was turned upside down 5 times and allowed to stand for 4 minutes to activate the whole blood. Finally, 20 μL of CaCl_2_ reagent and 340 μL of activated whole blood were added to the test cup and tested.

### 2.8. Antithrombotic properties of Schistosoma Kunitz peptide Shp4

Antithrombotic properties *in vivo* were determined using 10% FeCl_3_-induced carotid artery thrombosis. All animal experiments were carried out under a protocol approved by the Institutional Animal Care and Use Committee, Hubei University of Medicine. The FeCl_3_-induced carotid artery thrombosis model was established as described previously [34]. C57BL/6 male mice (9–11 weeks old, 25–30 g) were anesthetized with an intraperitoneal injection of 10% chloral hydrate (4 ml/kg). C57BL/6 mice (n = 18) were divided randomly into 3 groups of 6 mice each. Experiments were performed to evaluate the efficacy of Shp4 peptide (1.0 mg/kg) with a positive control heparin (3.6 mg/kg) and a vehicle control (normal saline, 0.154 M). Approximately 100 μL of drug was injected into the mice via the tail vein. The right carotid artery was exposed using blunt dissection, and vascular injuries were caused by applying filter paper of 2 mm × 1 mm saturated with 10% FeCl_3_ on top of the carotid artery for 3 min. After 3 min of FeCl_3_ exposure, the filter paper was removed, and the vessel was washed with sterile normal saline. After 15 min, mice were killed via cervical dislocation immediately after the conclusion of the experiment and prior to recovery from anesthesia. The right common carotid artery with the thrombus was exposed, ligated and separated. The length and wet weight of each thrombus were measured, and then the thrombi were fixed with 4% paraformaldehyde.

### 2.9. Statistical analysis

The results are expressed as the means ± SDs unless otherwise described. The curve of residue enzyme activity vs. inhibitor concentration was generated in SigmaPlot 12.5 software, and the IC_50_ was calculated according to the following equation (four parameter logistic curve): y = min+(max-min)/(1+(x/IC_50_)^-Hillslope^). Statistical differences among the animal groups were analyzed by unpaired t test.

## 3. Results

### 3.1. Molecular characterization of a common Kunitz-domain Shp4 from nine schistosoma-derived secreted proteins

**Fig 1:**
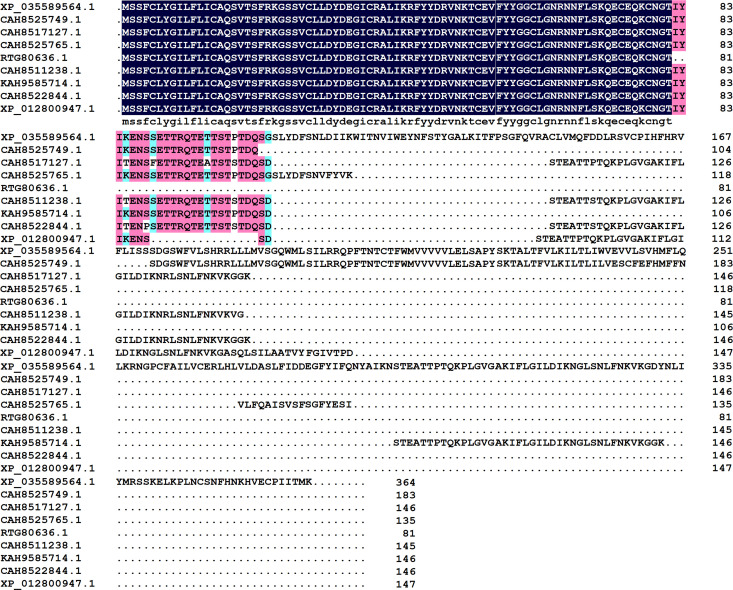
Molecular diversity and sequence alignments of nine Schistosoma secreted proteins with a common Kunitz-domain. The pink color means all the amino acids at this site was same, and the blue color means the amino acids at this site had over 75% similarity. XP_035589564.1, CAH8525749.1, KAH9585714.1 and XP_012800947.1 were from *Schistosoma haematobium*, CAH8517127.1 was from *Schistosoma guineensis*, RTG80636.1 and CAH8511238.1 were from *Schistosoma bovis*, and CAH8522844.1 was from *Schistosoma curassoni*.

By sequence blasting and structural fold searching of the transcriptomic profile and genomic data of 11 schistosoma species, available at WormBase ParaSites and NCBI [42,48], a common Kunitz-domain shp4 was discovered from nine schistosoma-derived secreted proteins, such as XP_035589564.1, CAH8525749.1, KAH9585714.1 and XP_012800947.1 from *Schistosoma haematobium*, CAH8517127.1 from *Schistosoma guineensis*, RTG80636.1 and CAH8511238.1 from *Schistosoma bovis*, and CAH8522844.1 from *Schistosoma curassoni*. Sequence alignments showed that all the nine secreted proteins adopt the common Kunitz-domain Shp4, but have different C-terminal non-Kunitz-domain fragments (Fig 1). Schistosoma species distribution of Kunitz-domain Shp4 showed that there are four schistosoma species *Schistosoma haematobium*, *Schistosoma guineensis*, *Schistosoma bovis* and *Schistosoma curassoni* that have the common Kunitz-domain Shp4, suggesting its possible important role in the survival of Schistosoma. So, in the next work we focused on the functional characterization of Shp4, because it is the common Kunitz-domain from nine schistosoma-derived secreted proteins and four schistosoma species.

**Fig 2:**
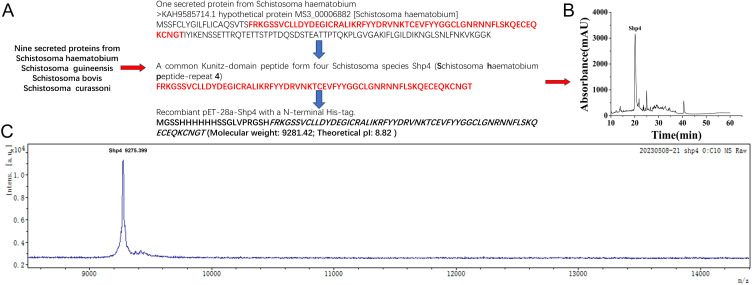
Expression and purification of the common Kunitz-domain peptide that was named Shp4. **A.** one representative secreted protein KAH9585714.1 of nine schistosoma secreted proteins from the *Schistosoma haematobium*. The common domain in nine schistosoma secreted proteins was characterized from KAH9585714.1 and named Shp4 (*Schistosoma haematobium* peptide-repeat 4). **B.** Expression and purification of the common Kunitz-domain peptide Shp4. **C.** Recombinant Shp4 was further tested by mass spectrometry. The measured molecular weight of Shp4 was 9275.399 Da, which was consistent with its calculated value of 9275.42 Da.

Shp4 was expressed with the expression vector pET28a that we have described before [30], which was found to exclusively accumulate in inclusion bodies. It was refolded in vitro at approximately pH 8.0 (0.2 M ammonium acetate at pH 8.0, containing 0.2 mM oxidized glutathione and 0.5 M arginine) at 16 °C for 24 h. The refolded oxidized Shp4 was separated by HPLC, and the fraction at approximately 21 min was collected. Recombinant Shp4 was further tested by mass spectrometry. The measured molecular weight of Shp4 was 9275.399 Da, which was consistent with its calculated value of 9275.42 Da (Fig 2). These results showed that recombinant Shp4 was prepared successfully and could be used for further functional evaluation.

### 3.2. Shp4 is a direct inhibitor toward coagulation factor XIa

**Fig 3:**
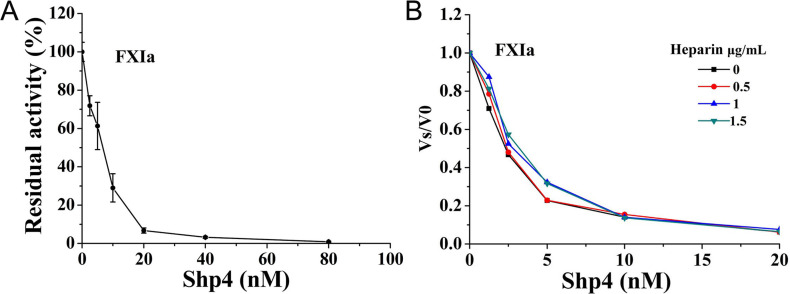
FXIa inhibitory activity of Shp4. **A.** Concentration-dependent curve of the FXIa inhibitory activity of Shp4. **B** Concentration-dependent curve of the FXIa inhibitory activity of Shp4 with different concentrations of heparin.

Coagulation factor XIa is a novel anticoagulant drug target, but no XIa inhibitors have been found from blood-sucking schistosoma, including the four species *Schistosoma haematobium*, *Schistosoma guineensis*, *Schistosoma bovis*, *Schistosoma curassoni* and others. Firstly, we tested the inhibitory activity of recombinant peptide Shp4 towards coagulation factor XIa. Excitingly, at the concentration of 20 nM, Shp4 have showed apparently inhibitory effects on coagulation factors XIa. Besides this, Shp4 inhibits XIa activities in a non-heparin-dependent manner [27], suggesting its direct inhibiting activity towards this target (Fig 3). Concentration dependent experiments showed coagulation factor XIa was significantly inhibited by Shp4 with an IC_50_ value of 7.10 ± 1.46 nM.

**Fig 4:**
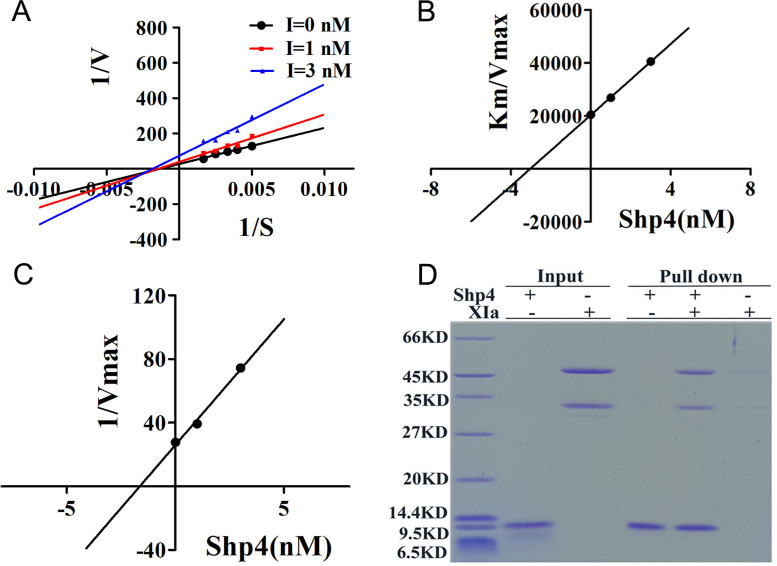
Enzyme kinetics and binding experiments of Shp4 towards coagulation factor XIa. **A.** Double reciprocal of 1/V to 1/S of Shp4 toward XIa. **B.** Km/Vmax for different concentrations of Shp4 toward XIa. **C.** 1/Vmax for different concentrations of Shp4 toward XIa. **D.** Shp4 binds XIa directly, as evaluated by His pull-down.

Next, enzyme kinetics experiments showed that the inhibition type of Shp4 on FXIa belongs to mixed inhibition, indicating that Shp4 can bind to both FXIa and FXIa substrate complexes. The Ki value that represents the binding ability of inhibitor towards free enzyme is 3.346 nM, and the Ki’ value that represents the binding ability of inhibitor towards free enzyme-substrate complex is 1.638 nM. Due to the fact that the Ki value is similar to the Ki’ value, we can conclude that the inhibition type of Shp4 towards XIa is non-competitive inhibition, a special form of mixed inhibition (Fig 4). Next, the His pull-down experiment was used to verify the direct interaction of Shp4 with coagulation factor XIa. In the His pull-down experiment, the Ni-beads bound the Shp4 peptide for its His-tag. If XIa can bind the Sh4 peptide directly, it will be pulled by the Ni-beads together with the Shp4 peptide. Our results indicated that Shp4 can directly bind XIa, which was consistent with the fact that Shp4 inhibited XIa activities in a non-heparin-dependent manner (Fig 4).

**Fig 5:**
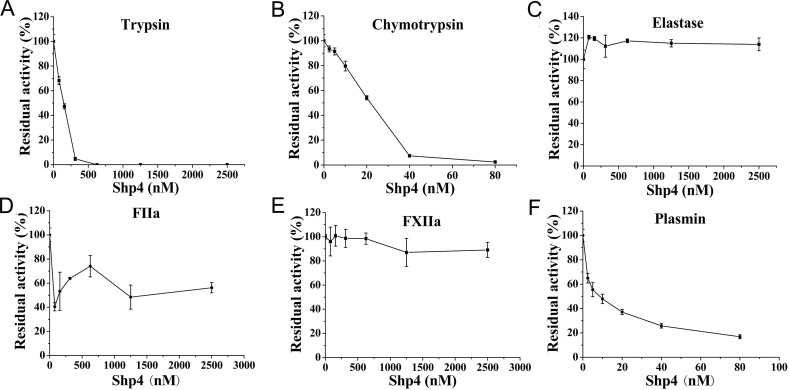
Inhibitory activities of Shp4 toward other serine proteases. **A.** Inhibitory effect of Shp4 on the serine protease trypsin. **B.** Inhibitory effect of Shp4 on the serine protease chymotrypsin. **C.** Inhibitory effect of Shp4 on the serine protease elastase. **D.** Inhibitory effect of Shp4 on the serine protease FIIa. **E.** Inhibitory effect of Shp4 on the serine protease FXIIa. **F.** Inhibitory effect of Shp4 on the serine protease plasmin.

In addition, at the same concentration of 20 nM, Shp4 had almost no inhibitory effects on another coagulation factors FXIIa and thrombin (FIIa), and had weak inhibitory activity towards plasmin, suggesting its potential application in the lead peptide drug development for XIa-specific new anticoagulants (Fig 5). In conclusion, Shp4 is a highly active anticoagulant with direct factors XIa binding activity and heparin concentration in-dependent inhibiting activity, and might be a lead drug for thrombosis-associated diseases. Besides this, Shp4 had apparent inhibitory activity towards digestive system serine proteases trypsin and chymotrypsin, but no inhibitory activity towards elastase (Fig 5), suggesting that Shp4 had a certain degree of protease tolerance.

### 3.3. Anticoagulant activity of Shp4 in plasma and whole blood

**Fig 6:**
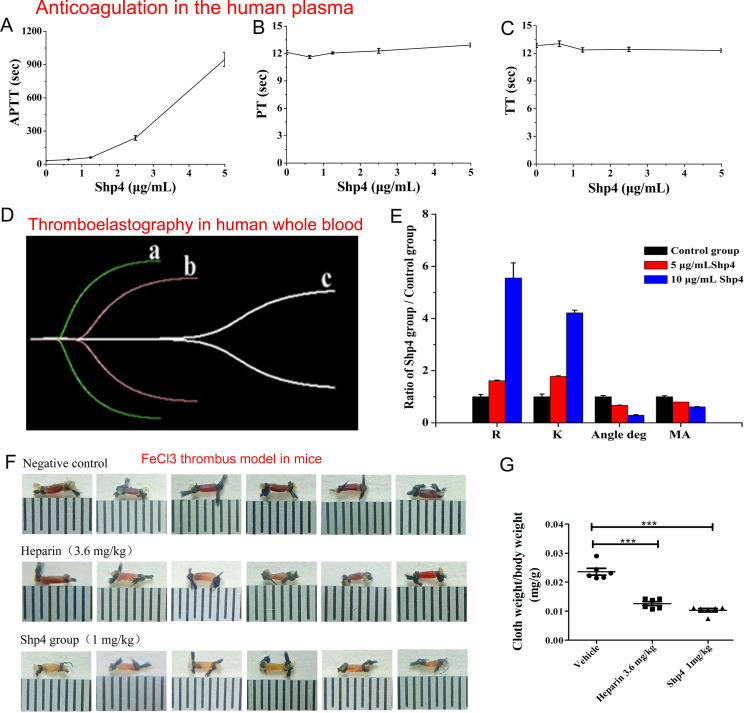
Anticoagulation functional characterization of the common Kunitz-type peptide Shp4 *in vitro* and *in vivo.* **A.** Concentration-dependent curve of Shp4 evaluated by APTT. **B.** Concentration-dependent curve of Shp4 evaluated by PT. **C.** Concentration-dependent curve of Shp4 evaluated by TT. **D.** Effects of different concentrations of Shp4 on whole blood coagulation, a. b and c represent the control group, and peptide Shp4 groups with concentrations of 5 μg/mL and 10 μg/mL, respectively; whole blood coagulation curve. **E.** The ratios of R, K, angle deg, and MA in the Shp4 and control groups. **F.** Antithrombotic effect of Shp4 in the FeCl3-induced right carotid artery thrombosis model in mice. Thrombosis was induced by 10% FeCl3, 2 mm × 1 mm filter paper; six mice per group. Images of common carotid artery thrombosis in the negative control group, 3.6 mg/kg heparin group and 1 mg/kg Shp4 group. **G.** Antithrombotic effect analyses of Shp4. The significant difference between the negative control group and the 1 mg/kg Shp4 group was calculated by unpaired t test, ***P < 0.001.

Schistosoma is a kind of blood-sucking animal that has a unique parasite life cycle. Schistosoma lives in the vein of the mammalian host and feeds on its blood, which suggests that schistosoma might be a natural source of novel anticoagulants. So, the anticoagulation activity of Shp4 was further performed with APPT, PT and TT assays. The results showed that Shp4 inhibited the APPT pathway with a concentration-dependent effect. When the peptide concentration increased to 5 μg/ml, Shp4 still had no apparent inhibitory activity toward the extrinsic coagulation pathway and the common coagulation pathway (Fig 6). These results showed that Shp4 is a potent and selective anticoagulant toward the intrinsic pathway, which was consistent with its specific XIa inhibitory activity.

To further evaluate the anticoagulation function of Shp4 in whole blood, thromboelastography was used to assess its effect. Thromboelastography showed that both 5 μg/mL and 10 μg/mL Shp4 had anticoagulation effects in whole blood, and the inhibitory effect of 10 μg/mL Shp4 was apparently stronger than that of 5 μg/mL Shp4 (Fig 6). Four statistical indices, R (representing clotting time), K and angle (reflecting clot strength and development, respectively), and MA (maximum platelet-fibrin clot strength), of thromboelastography were further analyzed. Generally, if the tested blood showed a hypercoagulable state, the TEG findings included a very low R (reaction time) and K (kinetics) and increased maximum amplitude (MA) and angle. In the presence of 5 μg/mL and 10 μg/mL Shp4, an apparent increase in R (reaction time) and K (kinetics) and a decrease in maximum amplitude (MA) and angle were found, which showed that Shp4 still had anticoagulant activity in whole blood, including all kinds of blood cells and blood platelets (Fig 6). Thromboelastography experiments showed that Shp4 is an effective anticoagulant in whole blood.

### 3.4. Antithrombotic effect of Shp4 *in vivo*

The Shp4 peptide showed good anticoagulation activity in vitro. To further evaluate its lead drug potential, antithrombotic effect was performed *in vivo*. In the presence of the positive control drug (3.6 mg/kg heparin), all six thrombus formations were decreased, and the blood vessels looked more transparent than those in the normal saline control group (Fig 6). The antithrombotic effect of 1 mg/kg Shp4 peptide was similar to that of the positive control group (3.6 mg/kg heparin), suggesting that schistosoma-derived Kunitz peptide Shp4 might be an effective anticoagulant. Statistical analysis of the cloth weight of the formed thrombus/body weight of the tested mice further confirmed the observational results (Fig 6). There were apparent differences between the shp4 peptide group and the normal saline control group (P < 0.001). These results showed that schistosoma-derived Kunitz peptide Shp4 can markedly restrain thrombus formation and reduce thrombus weight in the blood of living mice.

### 3.5. R18 is the common key residue of Shp4 for XIa inhibitory and anticoagulation activities

**Fig 7:**
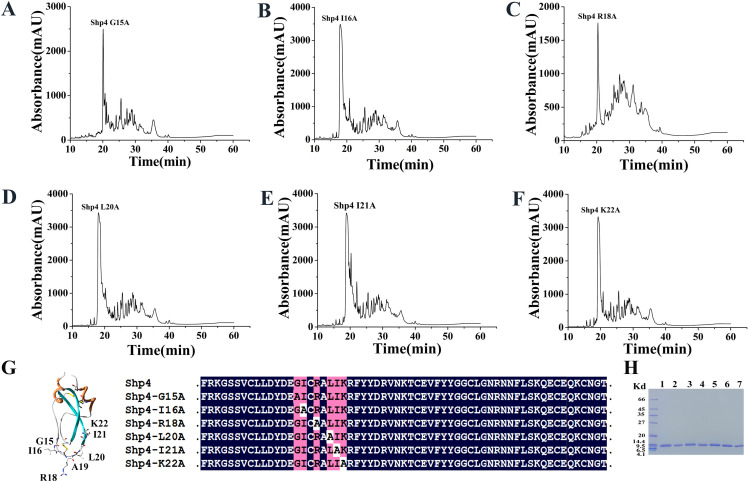
Recombinant expression and purification of six alanine scanning mutants of Shp4. **A.** HPLC purification of Shp4-G15A. **B.** HPLC purification of Shp4-I16A. **C.** HPLC purification of Shp4-R18A. **D.** HPLC purification of Shp4-L20A. **E.** HPLC purification of Shp4-I21A. **F.** HPLC purification of Shp4-K22A. **G.** Sequence alignments of six alanine scanning mutants of Shp4. **H.** SDS-PAGE characterization of six alanine scanning mutants of Shp4.

Shp4 is a classical Kunitz-type peptide, which has six cysteine and can form three conserved disulfide bridges C8-C41, C17-C54 and C33-C58. From the modeled 3-D structure of Shp4, we can recognize the serine protease-inhibitory loop P4-P4′ G15-I16-C17-R18-A19-L20-I21-K22. In order to confirm the function sites of Shp4 towards coagulation factor XIa and intrinsic coagulation pathway, alanine scanning strategy was used and six Shp4 mutant peptides were designed Shp4-G15A, Shp4-I16A, Shp4-R18A, Shp4-L20A, Shp4-I21A and Shp4-K22A. By the similar expression system with wild peptide Shp4, six mutant peptides were expressed successfully (Fig 7).

**Fig 8:**
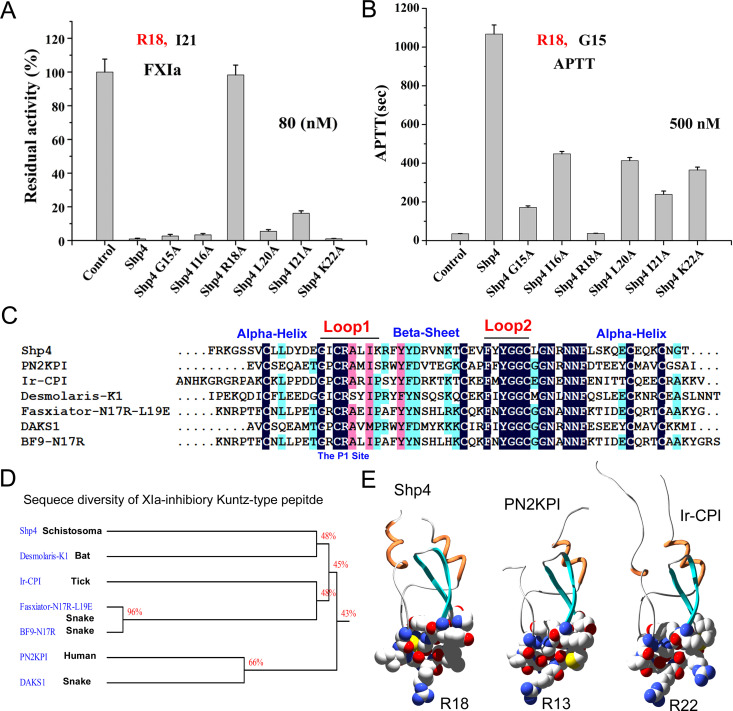
Structure-activity relationship of Shp4 and molecular diversity of XIa inhibitors with Kunitz-type structural fold. **A.** FXIa inhibitory of six alanine scanning mutants of Shp4. **B.** Anticoagulation activity evaluation of six alanine scanning mutants of Shp4. **C.** Sequence alignments of Shp4 with other reported XIa inhibitors with Kunitz-type structural fold. **D.** Phylogenetic analysis of Shp4 with other reported XIa inhibitors with Kunitz-type structural fold. **E.** The potential functional loops of three XIa inhibitors Shp4, PN2KPI and Ir-CPI.

Protease inhibitory experiments showed that the inhibitory activity of Shp4-R18A and Shp4-I21A were apparently weaker that Shp4, suggesting that these sites also have their contribution to the XIa inhibitory activity of the wild type peptide Shp4. Among them, R18 is the key residue for its XIa inhibitory of Shp4. APTT experiments showed that the anticoagulation activity of Shp4-G15A, Shp4-R18A and Shp4-I21A were apparently weaker than Shp4, suggesting that these sites have their contribution to the anticoagulation inhibitory activity of the wild peptide Shp4. Among them, R18 is the key residue for anticoagulation activity of Shp4 (Fig 8A and 8B). Together, the alanine scanning experiment indicated that R18 is the common key residue of Shp4 for XIa inhibitory and anticoagulation activities. Before our present work, some highly activity XIa peptide inhibitors with Kunitz-type structural fold have been discovered and designed, such as PN2KPI from human [49,50], Ir-CPI from tick [26], and Desmolaris-K1 from bat [27], Fasxiator-N17R-L19E, DAKS1, and BF9-N17R from snake [28,29,47]. Sequence alignments showed that although these Kunitz-domain peptides have potent XIa inhibitory and well anticoagulation activities, but their primary structure were very different, especially for Kunitz-domain peptides from different resource. However, when we analyse the conserved detailed regions from these XIa-inhibitory peptide, it was found that the conserved region majorly located at the functional loop1 and loop2 regions, but the non-conserved regions majorly located at the scaffold region to stabilize the whole structure of the Kunitz-domain peptides, such as alpha-helix and beta-sheet regions. The P1 site R is conserved in almost all the XIa-inhibitory Kunitz-type peptides. These conservations might mean that the optimization space of XIa-inhibitory Kunitz-type peptides in the classical loop1 and loop2 regions is very limited, and how to use the important lead drug scaffold Kunitz for the discovery and engineering of XIa inhibitors is still a huge challenge [10].

## 4. Discussion

The balance between the coagulation system and anticoagulation system by anticoagulants is still a challenge. Target-specific might be the key point, such as the classical specific inhibitors towards Xa and thrombin, and the novel inhibitors towards XIa [10–12]. The Kunitz-domain peptide is a classical scaffold that includes one antiparallel β-sheet and one or two α-helixes with diverse pharmacological activities [51–54]. Structural conservation of the Kunitz-type peptide and structural similarity of different serine proteases suggested that it might be very difficult to obtain specific Kunitz-type anticoagulants. In fact, although some potent XIa inhibitors have been found, but plasmin or Xa inhibitory activities might always influence their specificity. The discovery of coagulation factor XIa specific inhibitors with Kunitz-domain as a molecular scaffold is still a challenge, and whether to abandon the research on XIa inhibitory peptides with Kunitz-type structural fold is also a necessary issue that must be faced by researchers [28,49].

In order to deal with this awkward issue and promote the research and development work about peptide lead drug towards XIa, some pioneer scientists attempted to used target combination effects. Multiple targets or Target-specific might not be the main point, the right target or the right target combination might be the key point [16]. How to select and find the right target or the right target combination, it is still under research. Target characterization of new natural anticoagulants with good antithrombotic effects *in vivo* might provide new clue to the challenge, such as XIa from natural human anticoagulant PN2KPI [50], Xa and XIa combination from natural bat anticoagulant Desmolaris [27], and XIa and XIIa combination from natural tick anticoagulant Ir-CPI [55]. Our present work also indicated that schistosoma-derived XIa inhibitor Shp4 with classical Kunitz-type structural fold can markedly restrain thrombus formation and reduce thrombus weight in the blood of living mice, which was consistent the hypothesis that Target-specific might not be the only point, and Kunitz-type peptides with imperfect XIa specificity might also have its potential applications.

**Fig 9:**
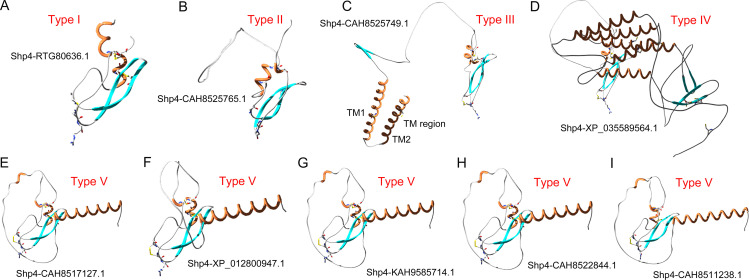
Classification of nine schistosoma secreted proteins based on their Kunitz-domains and C terminal regions. Type I includs the secreted protein RTG80636.1, Type II includes the secreted protein CAH8525749.1, Type III includes the secreted protein CAH8525749.1, Type IV includes the secreted protein XP_035589564.1, and Type V includes the secreted proteins KAH9585714.1, XP_012800947.1, CAH8517127.1, CAH8522844.1 and CAH8511238.1.

Besides this, the natural innovation of schistosoma Kunitz-domain containing secreted proteins provided new clues for us to control the Kunitz-domain by designing engineering specific C-terminal fragments, such as tethering Kunitz-domain peptide on the membrane by C-terminal TM fragments. As we know, schistosoma lives in the veins of the mammalian host and feeds on its blood, but no apparent hypercoagulability risk of blood has been reported in the process of their unique life cycle, suggesting that schistosoma might be a natural source of novel anticoagulants that can balance the coagulation and anticoagulation system [31]. In this study, we found nine schistosoma secreted proteins have a common Kunitz-domain, but different C- terminal fragments. Interestingly, by sequence blasting and structural fold searching of the transcriptomic profile and the genomic data of schistosoma, we found a common Kunitz-domain shp4 was discovered from nine schistosoma-derived secreted proteins. Functional characterization of the single Kunitz-domain Shp4 showed that it was a highly active anticoagulant with potent XIa inhibitory activity, but the full-length secreted proteins were very different. Interesting, all the non-Kunitz-domain fragments located at the C-terminal region of these nine secreted proteins. In order to explore the potential function roles of C-terminal fragments of these full-length secreted proteins, the 3-D structure of nine secreted proteins were further modelled using the SWISS-MODEL sever [38] (Fig 9).

Excitingly, besides the full-length proteins RTG80636.1, which is a Kunitz-type peptides, all the other eight full-length proteins have different C- terminal fragments. According to the structural characteristics, the nine secreted proteins from schistosoma can be divided into five types. Type I is a single Kunitz-type peptide without extra C-terminal fragments, including the secreted protein RTG80636.1. Type II has a short C-terminal fragment, which might have not apparent influence on the protease inhibitory activity of the N-terminal Kunitz-domain Shp4, including the secreted protein CAH8525749.1. Type III has a unique transmembrane region (TM region), which means that it might can be used to tether Kunitz-domain peptide on the membrane, including the secreted protein CAH8525749.1 [56,57]. Type IV has a very large C-terminal fragment, which might have might modulate and influence on the protease inhibitory activity of the N-terminal Kunitz-domain Shp4 by steric hindrance effect [58,59], including the secreted protein XP_035589564.1. Type V has a medium length C-terminal fragment, but it wrapped around the functional loop region of Kunitz-domain and also might have apparent influence on the protease inhibitory activity of the N-terminal Kunitz-domain Shp4, including the secreted proteins KAH9585714.1, XP_012800947.1, CAH8517127.1, CAH8522844.1 and CAH8511238.1. The natural innovation of schistosoma Kunitz-domain containing secreted proteins provided new clues for us to control the Kunitz-domain by designing engineering specific C-terminal fragments, such as tethering Kunitz-domain peptide on the membrane by C-terminal TM fragment and wrapping around the functional loop region of Kunitz-domain with a short loop [60,61]. So, C-terminal fragments might also be a novel strategy to control and influence the function of Kunitz-domain, such as tethering it on the membrane by the TM fragment, and wrapping around the functional loop region of Kunitz-domain with a short loop. Molecular design and engineering Kunitz-domain peptides with specific C-terminal fragments might be a new method to obtain functional and medicinal peptides with Kunitz-type scaffold.

In conclusion, our work found a specific Kunitz-domain Shp4 that appeared in nine secreted proteins four schistosoma species with potent XIa inhibitory activity, well antithrombotic effects. Besides this, by structural modelling of nine schistosoma-derived secreted proteins, we firstly found that the C-terminal non-Kunitz-domain fragments might play important roles in modulating the Kunitz-domain protease-inhibitory activities by steric hindrance effect and transmembrane helix structure.
